# Force Modeling, Identification, and Feedback Control of Robot-Assisted Needle Insertion: A Survey of the Literature

**DOI:** 10.3390/s18020561

**Published:** 2018-02-12

**Authors:** Chongjun Yang, Yu Xie, Shuang Liu, Dong Sun

**Affiliations:** 1 Department of Instrumental and Electrical Engineering, Xiamen University, Xiamen 361005, China; yangchongjun@stu.xmu.edu.cn (C.Y.); xieyu@xmu.edu.cn (Y.X.); 2 Shenzhen Research Institute of Xiamen University, Shenzhen 518000, China; 3 School of Mechanical and Power Engineer, East China University of Science and Technology, Shanghai 200237, China; 4 Department of Mechanical and Biomedical Engineering, City University of Hong Kong, Hong Kong 999077, China; medsun@cityu.edu.hk

**Keywords:** needle insertion, force modeling, force measurement, parameter identification, force control

## Abstract

Robot-assisted surgery is of growing interest in the surgical and engineering communities. The use of robots allows surgery to be performed with precision using smaller instruments and incisions, resulting in shorter healing times. However, using current technology, an operator cannot directly feel the operation because the surgeon-instrument and instrument-tissue interaction force feedbacks are lost during needle insertion. Advancements in force feedback and control not only help reduce tissue deformation and needle deflection but also provide the surgeon with better control over the surgical instruments. The goal of this review is to summarize the key components surrounding the force feedback and control during robot-assisted needle insertion. The literature search was conducted during the middle months of 2017 using mainstream academic search engines with a combination of keywords relevant to the field. In total, 166 articles with valuable contents were analyzed and grouped into five related topics. This survey systemically summarizes the state-of-the-art force control technologies for robot-assisted needle insertion, such as force modeling, measurement, the factors that influence the interaction force, parameter identification, and force control algorithms. All studies show force control is still at its initial stage. The influence factors, needle deflection or planning remain open for investigation in future.

## 1. Introduction

### 1.1. Background

Needle insertion is commonly used in modern surgery, especially in the field of minimally invasive surgery (MIS). Under the guidance of imaging technology or other detectors, flexible needles pass through soft tissue to complete MIS, administer local anesthetic drugs, sample blood, perform neurosurgery, achieve placement and brachytherapy, etc. [1,2,3,4,5,6,7,8,9,10]. Meanwhile, percutaneous bioscopy and therapy are widely used in the prostate, lung, liver, kidney, spine, and other organs and tissues [1,2,3,4,5,6,7]. Currently, the accuracy and quality of needle insertion depends entirely on the experience and ability of the surgeon because centesis has mainly been accomplished manually.

In the past decade, robotic-assisted manipulation has been increasingly recognized in modern medicine. Meanwhile, needle insertion is the first step for most MIS, and the most fundamental manipulation during MIS [1,2,3,4,5,6,7,8,9,10,11]. Physicians and patients anticipate a high-powered needle insertion surgery system with good reliability, high accuracy, rapid recovery, less pain, high satisfaction and low cost [1,2,3,4,5,6,7,8,9,10,11]. The successful use of robotic-assisted operation systems such as da Vinci and ZEUS has attracted considerable interest from the medical and engineering communities [7,11]. 

The da Vinci operation system is a master-and-slave system. Master-and-slave systems always use a force control strategy that uses position feedback [7]. Namely, the movement of the slave hand can be indirectly manipulated by handling the master hand with the help of visual position information. However, the operator cannot directly feel the operation because the surgeon-instrument and instrument-tissue interaction force feedbacks are lost [1,2,3,4,5,6,8,9,10,12,13,14,15,16,17,18,19]. Meanwhile, public experimental data indicate that the medical robot must contact several inhomogeneous tissues [1,2,3,4,5,6]. Therefore, advancements in force feedback and control not only reduce tissue deformation and needle deflection but also provide the surgeon with better control over the surgical instruments. 

Furthermore, when the surgical robot interacts with its environment by puncturing, twisting, cutting, etc., it is expected to autonomously identify the unforeseen circumstances and implement the appropriate algorithms [1,2,3,4,5,6,8,9,19,20]. Thus, there is a sharply increasing desire for force control research in the field of surgical robots in recent decades, especially for active force control [21]. 

### 1.2. Related Work

Previous studies have focused on needle steering, experimental data analysis, and the needle-tissue interaction mechanism [1,2,4,5,6]. However, the goal of this survey is to summarize the key components surrounding force control during robot-assisted needle insertion, including state-of-the-art force modeling, identification and control algorithms based on related studies. 

Abolhassani et al. [1] provided a review of the interaction forces, tissue deformation, needle deflection, and trajectory planning as well as their applications during needle insertion. Misra et al. [2] constructed a survey that emphasized the continuum mechanics and finite element modeling for a virtual reality simulation. Cowan et al. [4] provided an overview of the flexible needle steering technology including the design, modeling, planning and image guidance. Gerwen et al. [5] focused on experimental data classification to analyze the correlation between insertion force and the factors that influence it. Elgezua et al. [6] performed a survey of the limitations and challenges of needle trajectory calculations and vision-based needle navigation for minimally invasive treatments.

### 1.3. Aims and Methods

Force control plays a major role in the enhancement of human-robot interaction realization and the achievement of high-performance robotic systems during needle insertion. To better understand the force information acting on a needle during insertion and aid researchers in this field, we surveyed the literature and conducted a general review of some critical topics related to force control during needle insertion to help achieve needle insertion force control. 

This survey of the literature is limited to articles related to force modeling, identification, measurement, and control methods during needle insertion into different tissues or materials. For example, some typical search terms are used such as “needle insertion force” with “modeling”, “measurement”, “identification”, or “control”. The search was conducted during the middle months of 2017 using the PubMed database, the Web of Science and Google Scholar. Relevant articles were found by considering the title, key word, abstract, and conclusion. The tables, figures and other details on available information were then further investigated. In addition, references found in these papers were studied. This leads to a total of 166 papers, grouped by topic, as shown in Table 1. 

**Figure 1:**
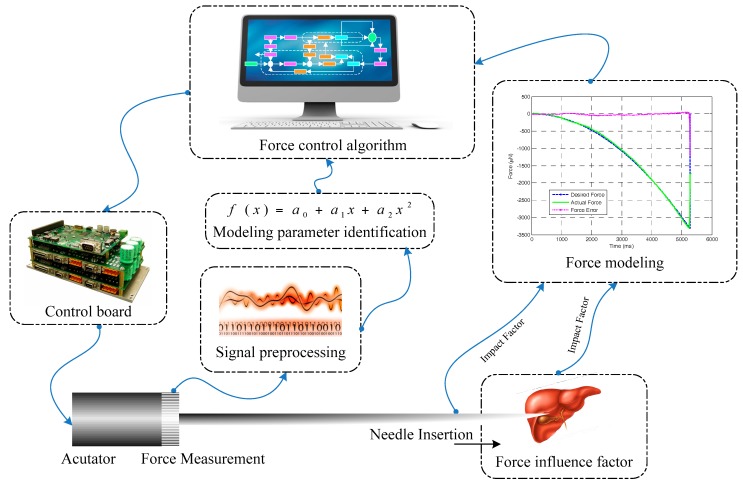
Schematic of the structure of the survey.

According to the groups, this paper is organized as follows. In Section 2, we focus on the force modeling of needle insertion into soft tissue. After that, an overview of force measurement methods during needle insertion is presented in Section 3. The influence factors and parameter identification methodology for force control during needle insertion are discussed in Section 4 and Section 5, respectively. In Section 6, we survey and provide an outlook for robot-assisted needle insertion force control methods. Finally, the current state of the art is summarized, and an outline of future directions is shown. Figure 1 illustrates the scope of this survey. 

## 2. Methods of Needle Insertion Force Modeling

In past decades, scholars have presented a series of works focusing on force modeling to better understand the needle-tissue interaction in theory and practice [4,12,14,15,17,22,23,24,25,26,27,28,29,30,31,32,33,34,35,36,37,38,39,40,41,42,43,44,45,46,47,48,49,50,51,52,53,54,55,56,57,58,59]. In this section, we classify the needle insertion force modeling into the following categories: finite element methods (FEM) [4,29,31,40,44,47,49], energy methods [17,38,39,59], statistical methods [27,46] and analytical methods [12,14,15,22,23,24,25,26,28,29,30,31,32,33,34,35,36,37,43,45,48,50,52,53,54,55,56,57,58,114]. From the application of needle insertion, the FEM, energy, statistical and analytical methods all could be used in needle deflection, tissue deformation, path planning/navigation or force analysis. But from the point of robot force control, only the modeling using analytical methods is the most suitable candidate for online identification and control.

The FEM are featured with accurate representation of complex geometry, inclusion of dissimilar material properties and capture of local effects. But they need excessive calculation and the high precision of FEM mostly relies on their inputs. The energy methods calculate energy variation from deformation and easily represent the total solution. They are available for complex motion forms and neglect the specific process. But they do not reflect online detail information. The statistical methods could acquire data distribution characters and reflect patient-specific and procedure-specific criteria. However, they need data correlation analysis and require high integrity and offline estimation with huge workload. The analytical methods could reflect force information totally in detail and are not limited to their boundary conditions. They also have advantages with fast computation and online estimation. The typical features and applications of the modeling are summarized in Table 2. From the analysis of the table, we could find that modeling with analytical methods is the most suitable candidate for online force control. We thus focus on analytical method modeling in detail as follows. The needle insertion force is usually divided into stiffness force, friction force and cutting force [22,23,25,31,32]. To accurately express the tissue-needle interaction mechanism [22], the total applied force of the needle can be expressed by:

(1) $$f_{total0}(z)=f_{stiffness}(z)+f_{friction}(z)+f_{cutting}(z)$$

where *z* is the position of the needle tip, *f_total_* is the total axial force during needle insertion, and *f_stiffness_*, *f_friction_*, and *f_cutting_* represent the stiffness, friction and cutting force acting on the needle, respectively. The stiffness force occurs because the needle attempts to pierce the membrane. The friction force acting along the length of the needle could be caused by tissue adhesion and damping. The cutting force is necessary to slice the intact tissue to make the needle pass through the tissue [1,22]. 

**Figure 2:**
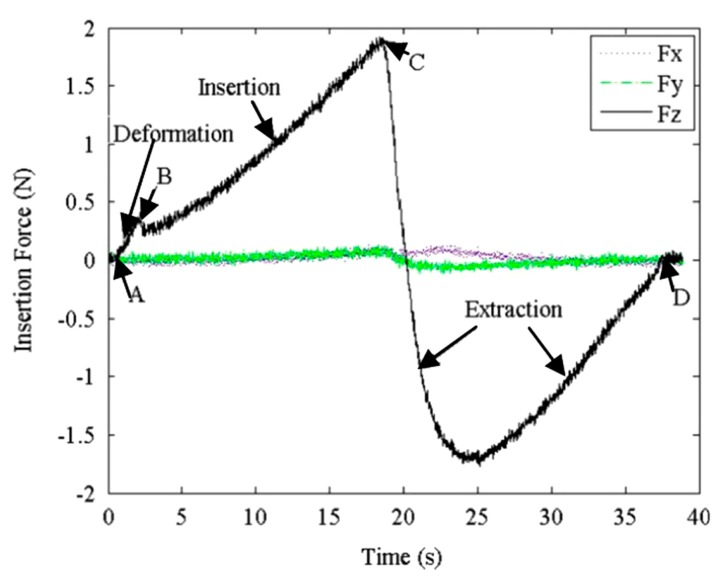
A force profile of needle-tissue interaction forces at 3 mm/s [25].

**Figure 3:**
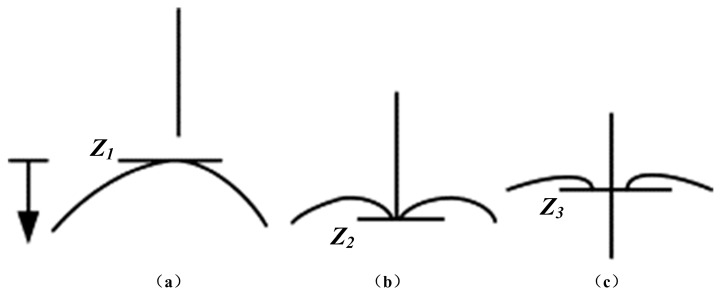
Locations of the tissue surface in different puncture stages: (**a**) pre-puncture *Z_1_*, (**b**) puncture *Z_2_*, and (**c**) post-puncture *Z_3_* [23].

Based on this theory, Okamura et al. [22] considered two basic phases of needle penetration: pre-puncture and post-puncture. Similarly, Jiang et al. [25] distinguished three basic phases of insertion: deformation, insertion and extraction, as shown in Figure 2. During the deformation phase (from A to B), the force steadily increases to a peak value after touching the surface of the tissue and sharply drops when the insertion event occurs. In this phase, the insertion force is equal to the stiffness force. In the insertion phase (from B to C), the force can be calculated by the friction and cutting force when the needle penetrates into soft tissue. When the needle is extracted from soft tissue, there is an extraction phase (from C to D) after the deformation and insertion phases; the extraction force is equal to the friction force, and there is no new cutting in this phase. Fx and Fy are the forces acting on the needle perpendicular to the axial force Fz [25]. These forces can be ignored in force modeling due to their small magnitudes compared to the axial force, as shown in Figure 2. The locations of the tissue surface at different insertion stages are shown in Figure 3. 

Based on the ideas initially proposed by Okamura et al. [22], many scholars further focused their studies on modeling the stiffness, friction, and cutting force. The typical features and applications of analytical methods are summarized in Table 3.

### 2.1. Stiffness Force Modeling

Simone [23] used a nonlinear spring model [24] in the human thigh deformation modeling to describe the nonlinear force caused by large deformations before insertion:

(2) $$f_{stiffness1}(x)=\frac{x}{ax+b}$$

where *x* is the length of the springs from their original to final positions, and *a* and *b* are determined by experimental data. From the experimental data, they found that the several-order polynomial model had lower root mean square (RMS) error values than the nonlinear spring model *f_stiffness_1*(*x*). Considering the insignificant RMS error variations of high orders, they modeled the stiffness force with a quasi-static model as follows:

(3) $$f_{stiffness2}(x)=\left\{ \begin{array}{ll} 0 & x_{tip}<x_{z1}\\ f(x) & x_{z1}\leq x_{tip}\leq x_{z2}\\ 0 & x_{tip}>x_{z3} \end{array}\right.$$

(4) $$f(x)=a_{0}+a_{1}x+a_{2}x^{2}$$

where *x* is the position of the needle tip, *f*(*x*) is a nonlinear spring stiffness model, *a_i_* (*i* = 0,1,2) are determined by experimental data, and *x_tip_*, *x_zi_* (*i* = 1,2,3) are the positions of the needle tip and tissue surface relative to a presupposed coordinate system as shown in Figure 3. Obviously, the specific offline parameters of this model were used only for corresponding conditions, and the offline tissue library was expected to be established in an all-round way. Therefore, online estimation of the model is expected to be constructed considering workload reduction. 

Similarly, considering the penetration depth and neglecting small motions between two objects, Barbé et al. [37] determined that the Hunt-Crossley model could match the deformation caused by needle insertion well and found a stiffness force model that varied in a nonlinear way as follows:

(5) $$f_{stiffness3}(x)=\left\{ \begin{array}{ll} -(\mu x^{n}+\lambda x^{n}v) & x>0\\ 0 & x\leq 0 \end{array}\right.$$

where *x* is the position of the tissue surface relative to a fixed coordinate system, *v* is the speed of the needle tip, and *μ*, *λ*, and *n* are constant parameters that depend on the material properties.

Based on the work of Maurel [52], Maurin et al. [28] described the insertion force model as an exponential function of the depth to make a comparison with the model *f_stiffness_1*(*x*) as follows:

(6) $$f_{stiffness4}(x)=(F_{0}+b)e^{a(x-d_{0})}+b$$

where *x* is the position of the needle tip and the parameters *F*_0_, *a*, *b*, and *d*_0_ are determined using experimental data and Newton-Raphson optimization. They found that the model more precisely described the force model than the second-order polynomial. However, the exponential function needed excessive calculation from the view of force control compared to the second-order polynomial model. 

**Figure 4:**
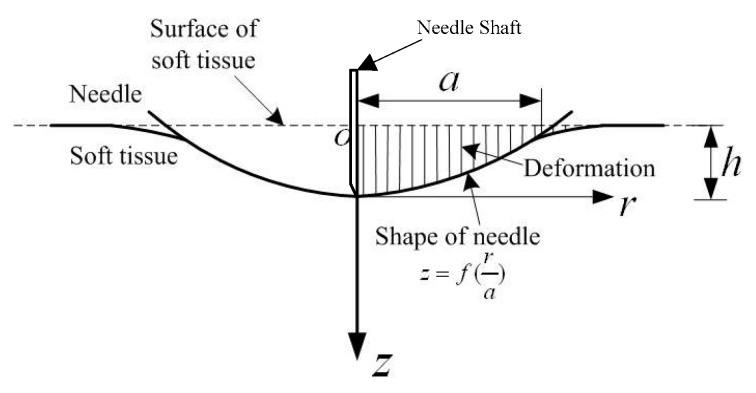
Contact mechanics model for stiffness force [25].

From another view of force modeling, Jiang et al. [25] held that the skin viscoelasticity and the elastic properties of the organ capsule during needle insertion could produce stiffness force, as shown in Figure 4. They therefore presented a contact model considering mechanical properties and deformation. They then applied Hankel transforms and the theory of dual integral equations into solving the contact mechanics. Based on the model of Sneddon [26], the expressions can be derived from the elementary solution:

(7) $$h=\int_{0}^{1}\frac{f^{\prime }(x)}{\sqrt{1-x^{2}}}dx$$

(8) $$f_{stiffness5}(x)=2E_{r}a\int_{0}^{1}\frac{x^{2}f^{\prime }(x)}{\sqrt{1-x^{2}}}dx$$

where *a* is the radius of the contact circle, *r* = *ax*, *r* ≤ *a*; *h* is the distance from the initial to the final position when the needle tip penetrates into the soft tissue; *E_r_* represents the reduced modulus calculated from the Young’s modulus of the needle and soft tissue; *f*(*x*) is the curve function of the needle referring to the needle tip as the original position; *x* is the needle position; and 0,1 is the integration range. In this model, they successfully avoided the influence caused by special needle and material properties. However, the method is unsuitable for realtime force control due to the unavailability of the online estimation methods for the model. 

### 2.2. Friction Force Modeling

**Figure 5:**
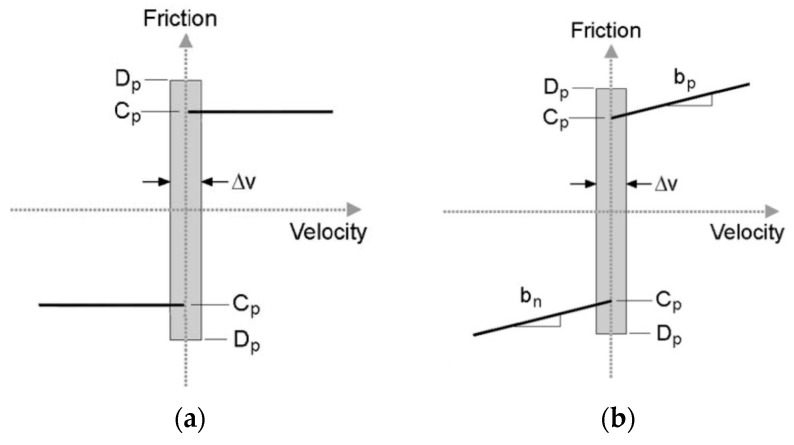
(**a**) Karnopp and (**b**) modified Karnopp friction models [23].

Simone [23] modeled friction force with a modified Karnopp model [53]. This model is based on the position sensing at low velocities and stick-slip friction as shown in Figure 5b.

(9) $$f_{friction1}(\dot{x},F_{a})=\left\{ \begin{array}{cc} C_{n}\mathrm{sgn}(\dot{x})+b_{n}\dot{x} & \dot{x}\leq -\Delta v/2\\ \max(D_{n},F_{a}) & -\Delta v/2<\dot{x}\leq 0\\ \min(D_{p},F_{a}) & 0<\dot{x}<\Delta v/2\\ C_{p}\mathrm{sgn}(\dot{x})+b_{p}\dot{x} & \dot{x}\geq \Delta v/2 \end{array}\right.$$

where *C_n_* and *C_p_* are negative and positive dynamic friction values, respectively; *b_n_* and *b_p_* are negative and positive damping coefficients, respectively; *D_n_* and *D_p_* are negative and positive static friction coefficients, respectively; $\dot{x}$ is the relative velocity of the needle and tissue; Δv/2 is the threshold value considering the velocity to be zero or not; and *F_a_* is the sum of non-frictional forces applied to the system. As shown in Figure 5a, the classical Karnopp model reflected the dynamic friction and static friction within a “dead zone” near zero velocity. Furthermore, the modified model successfully improved the classical Karnopp model with advantages of capturing the subtle effects of the Stribeck effect and Dahl model in soft tissue. 

**Figure 6:**
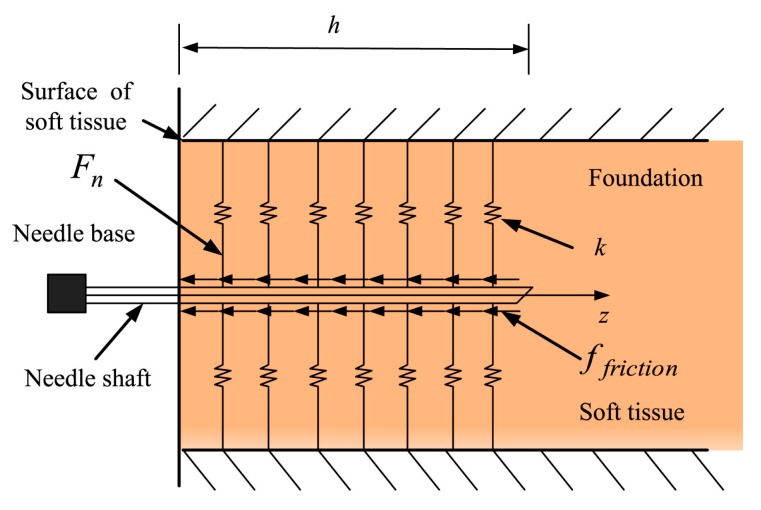
Modified Winkler’s foundation model for the friction force [25].

Small deformation of soft tissue occurs in the direction vertical to the needle shaft during needle insertion, which affects the measurement of relative velocity. In contrast, the needle-tissue axial forces are relatively uniform along the needle shaft according to the force distribution [29]. Thus, Jiang et al. regarded the distributed force along the needle as a modified Winkler’s foundation [30] with a linear stiffness coefficient as shown in Figure 6.

(10) $$F_{n}=kh\Delta$$

where *F_n_* is the normal force along the needle shaft due to the tissue deformation, Δ is the settling amount, *k* is the foundation modulus, and *h* refers to the contact length. The friction force acting along the needle shaft in the axial direction could then be viewed as the Coulomb friction and is given as:

(11) $$f_{friction2}=\mu F_{n}$$

where *μ* is the needle-tissue friction coefficient. 

The relative movement between the needle and the tissue is often invisible. Thus, it is difficult to obtain and estimate the criteria of the friction models. Yang et al. [31] presented a force model with several dominant frequencies due to some wave vibrations in experiments. The friction force is calculated using the Fourier series instead of the classical model in the time domain to avoid the difficulty of obtaining the needle-tissue relative velocity information:

(12) $$f_{friction3}=\sum_{i=0}^{M}\left[A_{i}\cos(\omega_{i}x)+B_{i}\sin(\omega_{i}x)\right]\;x>d$$

where *A_i_* and *B_i_* are Fourier coefficients determined from the experimental data of the needle insertion, *ω_i_* represents the truncated frequencies of vibratory friction force, *M* is the number of truncated frequencies, *d* is the needle displacement to penetrate the tissue, and *x* is the needle displacement.

On the other hand, the main parameters show only the static force or friction in the modified Karnopp model presented in [23]. However, when the needle passes through the tissue, the Karnopp model may not work because of significant presliding displacement in a dynamical condition. For this reason, Yang et al. [32] presented an Elasto-Plastic friction model by both friction and presliding displacement based on the study of Dupont [33]. The friction model can be shown as follows:

(13) $$\left\{ \begin{array}{ll} f_{friction4}=\sigma_{0}z+\sigma_{1}\dot{z}+\sigma_{2}\dot{x}\;\sigma_{j}>0 & j=0,1,2\\ \dot{z}=\dot{x}{\left(1-\alpha (z,\dot{x})\frac{\sigma_{0}}{f_{ss}(v)}\mathrm{sgn}(\dot{x})z\right)}^{i} & \frac{\sigma_{0}}{f_{ss}(\dot{x})}>0 \end{array}\right.$$

where *x* is a geometrical measurement of the interpenetration; *z* is the state of strain in the frictional contact; *v* is the collision speed; *σ*_0_ and *σ*_2_ are the Coulomb and viscous friction parameters, respectively; *σ*_1_ is the damping coefficient for the tangential compliance; *i* is an empirically determined parameter; *f_ss_*(*v*) is the Stribeck curve reflecting the steady-state friction force versus rigid body velocity; and *α*(*z,*$\dot{x}$) is used to achieve friction. 

Regarding the dynamical condition, Kobayashi et al. provided a friction force model based on the relative velocity between the needle and soft tissue instead of the absolute velocity to focus on the relationship between the friction and the velocity. The model developed from the experimental results can be expressed as follows:

(14) $$f_{friction5}=\left\{ \begin{array}{ll} A\ln(v)+B+Cvt & \left(v<1.5\mathrm{mm}/s\right)\\ F_{s}+Cvt & \left(v\geq 1.5\mathrm{mm}/s\right) \end{array}\right.$$

where *A* and *B* are parameters to be determined by the statistics, *v* is the relative velocity between the liver tissue and the needle, *C* is a parameter reflecting the slope of the line in the experiments, *F_s_* is the initial friction force with low-velocity characteristics, *t* is time, and 1.5 mm/s is regarded as a threshold value to distinguish high or low relative velocities.

To calculate the cutting force from the total measured force, Simone and Okamura [54] also found the friction force determined by:

(15) $$f_{friction6}=b_{p}lv_{needle}$$

where *b_p_* is the damping coefficient per unit length, which corresponds to a slow insertion speed; *l* is the length of the needle in the tissue; and *v_needle_* is the velocity of the needle tip.

Stellman [56] calculated the friction force by considering both the thickness and the elastic modulus of the material as follows:

(16) $$f_{friction7}=\mu E_{f}t_{f}d$$

where *μ* is the friction coefficient, *E_f_* is the material’s elastic modulus, *t_f_* is the thickness of cut, and *d* is the opening horizontal distance. 

**Figure 7:**
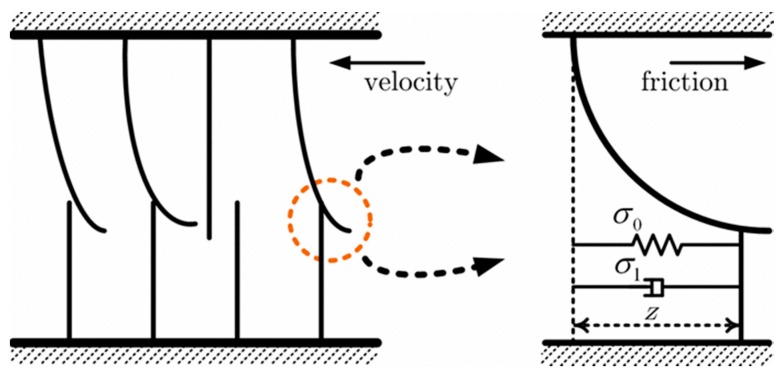
Microscopic representation of irregular contact surfaces and elastic bristles whose bending gives rise to the friction force [42].

Asadian et al. [55] provided a distributed LuGre model to present the dynamic characteristics along the needle to better understand the frictional behaviors during needle insertion. The basic principles are shown in Figure 7, where *z* is the supposed effect distance, *σ*_0_ is the stiffness coefficient of the microscopic deformations during the pre-sliding displacement, and *σ*_1_ is the damping coefficient associated with $\dot{z}$. In references [42,43,45,55], Asadian et al. found a series of modified LuGre models such as a static LuGre-based structure, a lumped model and a distributed dynamic nature. Meanwhile, they presented a method with a combination of the velocity estimator and the friction model to compensate for the velocity of the tissue with a needle shifting in the insertion direction. In this way, the friction-velocity cycle considering soft tissue deformation could be corrected to accurately model the friction force. 

Elgezua et al. [50] provided a method to acquire the patterns of needle-tissue interaction, which focused on the nonlinear local elastic modulus and real-time friction condition during insertion. They used a simplified friction force model to describe the small variation of the local force and deformations as follows:

(17) $$f_{friction8}=\frac{F_{k_{\min}}-F_{k0}}{x_{k0}-x_{k_{\min}}}(x-x_{k0})$$

where $F_{k_{\min}}$ is the force measured when the next insertion starts; *F*_*k*0_ is the force when the *k*-th insertion begins; *x*_*k*0_ and $x_{k_{\min}}$ are the needle positions when the *k*-th insertion starts and ends, respectively; and *x* is the needle position. However, as the authors presented in the work, the friction fitting model paid little attention to vein insertion with a double peak shape, and the calculated values of friction force were higher than the actual values.

Carra and Avila-Vilchis [36] selected the Dahl model [163] to represent friction with advantages of presliding displacement capture and viscous friction description in low-velocity regimes. The force model was given as:

(18) $$\left\{ \begin{array}{l} f_{friction9}=\int (\frac{df_{f}}{dx})dx+b\dot{x}\\ \frac{df_{f}}{dx}=\left\{ \begin{array}{ll} \sigma {\left|1-\frac{f_{f}}{D_{p}}\mathrm{sgn}(\dot{x})\right|}^{i}\mathrm{sgn}\left(1-\frac{f_{f}}{D_{p}}\mathrm{sgn}(\dot{x})\right) & \dot{x}>0\\ \sigma {\left|1-\frac{f_{f}}{D_{n}}\mathrm{sgn}(\dot{x})\right|}^{i}\mathrm{sgn}\left(1-\frac{f_{f}}{D_{n}}\mathrm{sgn}(\dot{x})\right) & \dot{x}<0 \end{array}\right. \end{array}\right.$$

where *x* is the position of the needle tip, $\dot{x}$ represents the velocity of the needle tip, *σ* is the slope of the friction curve at *f* = 0, and *i* is an empirically determined parameter that adjusts the shape of the friction slope function. *D_p_* and *D_n_* are the friction limits in the positive and negative directions, respectively; *f_f_* is friction force; and *b* is a viscous damping term. In this work, the Dahl model is simplified using *i* = 1. This model can predict the friction lag with the help of a partial differential equation (PDE). However, this model could not capture the Stribeck effect and reflect the static friction. 

### 2.3. Cutting Force Modeling

The cutting force is the force necessary to pass through the tissue. In [54], Simone and Okamura obtained the cutting force value by subtracting the estimated friction force from the total force after insertion:

(19) $$f_{cutting1}=\left\{ \begin{array}{cc} 0 & x_{tip}\leq x_{z2},t<t_{p}\\ C & x_{tip}>x_{z3},t\geq t_{p} \end{array}\right.$$

where *C* is a constant for a given tissue; *x_tip_*, *x*_*z*2_, and *x*_*z*3_ are the positions of the needle tip and the tissue surface relative to a fixed coordinate system before insertion; *t* is time; and *t_p_* is the time of insertion (B) as shown in Figure 2. However, the measured cutting forces with a slight increase were not quite constant due to internal collisions causing additional stiffness forces that should not be considered. The cutting force is theoretically viewed as stable constant in both tissues and phantoms at various insertion velocities with some fluctuations because the rupture was relying on the level of inhomogeneity of the object [12,22].

To better understand the real-time force property during insertion, Elgezua et al. [50] presented a cutting force modeling based on the supposition that the force acquired during the deformation phase was equal to the cutting force, as follows:

(20) $$f_{cutting2}=K_{kL}(x-x_{k0})+K_{kNL}(x-x_{k0}{)}^{2}$$

where *x* is the needle position, *x*_*k*0_ is the position at which the *k*-th insertion starts, and *K_kL_* and *K_kNL_* are the linear and nonlinear elastic moduli for the *k*-th insertion, respectively. 

Stellman [56] provided a maximum cutting force model by considering the effects of the contact areas and resistances. The contact areas were determined by the tip characteristics, and the resistances reflected the tearing or puncturing. The model was given as:

(21) $$f_{cutting3}=\frac{G_{p}A_{t}}{t_{field}}+\frac{G_{c}A_{c}}{d_{f}}$$

where *G_p_* represents the insertion resistance of the materials, *G_c_* reflects the tear resistance of the media, *A_t_* is the tip contact area, *A_c_* is the surface area of the tear or crack, *t_field_* is the field thickness, and *d_f_* is the position where the crack force is acting. 

### 2.4. Axial Insertion Force Modeling

In addition to classic stiffness-friction-cutting force model (Equation (1)), some scholars studied the axial insertion force as a whole without decomposition [15,45,57,58,162]. The soft tissue is boiled down to viscoelastic materials, and the insertion force could be calculated by the function of time-varying deformation accompanied by transformation of force and energy. Namely, the magnitude of the force acting on the needle equals the force on the interaction materials. The force is regarded as a combination of classical springs and dampers representing the elasticity and viscosity, respectively [15,57,58,162]. We summarize the common force modeling based on springs and dampers as shown in Table 4. Compared with the stiffness-friction-cutting force model (Equation (1)), the spring-damp force models cannot accurately reflect the true process of needle-tissue interaction. But they are easily used for online force estimation and control considering the simple model equation.

To efficiently manipulate forces by a simple model in computation and prepare it for real-time applications, Asadian et al. [45] established a force model with the use of nonlinear dynamics based on a modified LuGre model covering all phases of needle-tissue interaction including all forces acting on the needle. There is no separation between the axial force components and the entire intervention. Meanwhile, the method accurately reflects the real processing of the whole interaction and could be estimated online. Therefore it is available for real-time force control.

### 2.5. Multilayer Insertion Force Modeling

**Figure 8:**
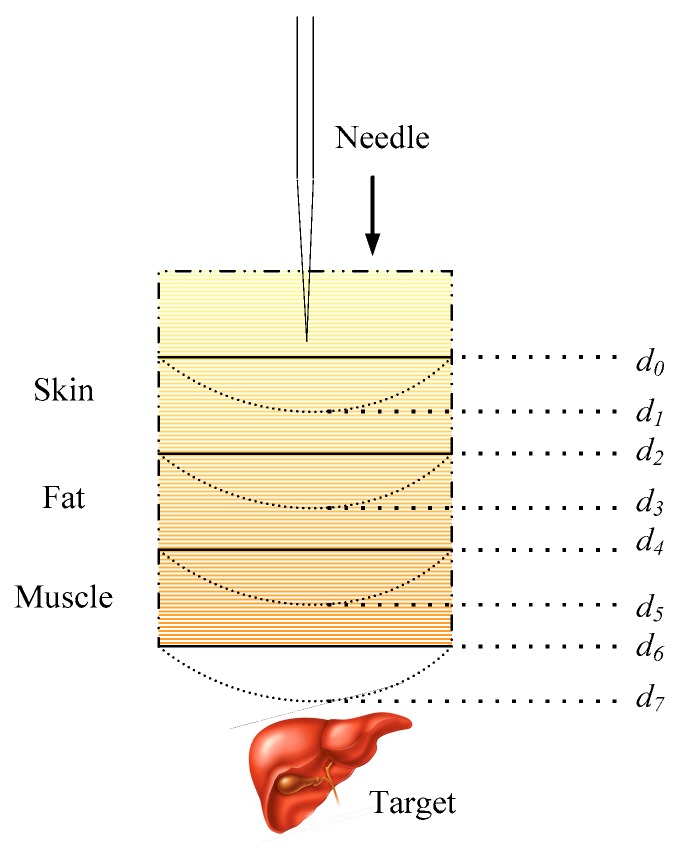
Needle insertion through several tissue layers.

After a series of force analyses of homogeneous materials and tissues in a single layer, some scholars focused on multiple layers or different materials and tissue modeling and simulation [14,36,48,114]. Typically, Carra and Avila-Vilchis [36] provided a new universal force model to study the needle insertion into several tissue layers such as skin, fat and muscle as shown in Figure 8. The complete force model included a nonlinear stiffness force model, a modified Dahl friction and a constant cutting. Li et al. [114] employed a contact model calculating the force–deformation response of a needle in contact with soft tissue. Gordon et al. [48] used an exponential function applied in a piecewise manner over the length of an entire needle insertion to acquire force. Heng et al. [14] provided a haptic simulator specialized for Chinese acupuncture learning and training. In this work, the bidirectional force model was analyzed and established by a combination of classical force models both statically and dynamically in different tissues such as skin, adipose tissue, muscle, and bone. These models described the force properties change of the needle when it goes through different tissue layers. Compared with the previous presented single layer models, the multilayer models are more practical in the needle insertion modeling of the whole robot-assisted MIS.

In short, we summarized the common force modeling methods from various perspectives in this Section. The available models can be selected in accordance with the characteristics of the proposed force control. In the future, new force modeling methods will be established to simplify the control algorithm with higher accuracy and shorter delay, reduce the hardware cost incurred by the control scheme, and improve the systematic stability and reliability.

## 3. Needle Insertion Force Measurement

After the analysis of the force modeling techniques, needle insertion force measurement and feedback must be considered in force control. In this section, we classify the force measurement and estimation methods considering existing technologies and design both directly and indirectly. The outlook of this section is shown in Table 5.

### 3.1. Direct Needle Insertion Force Measurement

The common direct methods for force sensing during needle insertion with a commercial or homemade force sensor include strain gauges, piezoelectric sensors and optical sensors [27,97,101]. 

Strain gauges are the most adopted sensing method to acquire the force acting on a needle due to tissue deformation during needle insertion [12,15,16,27,28,29,40,46,69,72,79,81,83,86,91,93]. Strain gauges are selected for their good performance with small size, fine sensitivity, easy multi-axis measurement and high strength during force sensing [41,50,60,62,74,76,80,81,82,84,87,88,89,90,92,95]. Drift and hysteresis are very challenging due to temperature changes and electromagnetic noise [100]. The ATI Nano and Mini series are the most selected sensors in the area of needle insertion owing to their excellent functions, which were widely used in [15,27,28,40,41,43,49,86,116]. The ATI series sensors could be sterilized with ethylene oxide or formaldehyde to ensure the security of the surgery [3,103]. This is because it is difficult to produce a sensor that endures the sterilization condition of HPHT [3,103]. In addition, some scholars focused on homemade strain gauges based on requirements for a unique purpose such as specially shaped structures, indenters and microneedle applications [66,88,95].

Piezoelectric sensors are other commonly used sensors for needle insertion. They are based on piezoelectric materials, including piezoelectric ceramics (PZT) and polyvinylidene fluoride (PVDF) [3,101,107,164]. In these materials, we could detect a voltage variation from the material caused by mechanical stress [3,101]. Piezoelectric sensors are famous for their large force measurement ranges, high bandwidths, small sizes and high power densities [101,107]. However, they are appropriate for dynamical, but not static, force measurements because of the drifting signal resulting from temperature changes and charge leakages [100].

During needle insertion, many authors presented optical sensors to measure force or pressure information based on the varying intensity or phase from a light signal [63,64,93,94,96,97]. An optical sensor is able to acquire force with multiple DOFs and in high-intensity magnetic fields for imaging with high sensitivity, reproducibility and no hysteresis [136]. However, optical sensors are limited to cable deformation or calibration and insensitive to small deformations caused by microforces [100]. In optical sensors, fiber Bragg grating (FBG) is popular in needle insertion, especially in ultra-precise applications such as retinal vein cannulation (RVC) [96,97,103]. 

There are some other direct technologies [106] used in needle insertion, such as capacitive-based [105] and resonance-based sensing methods. These methods were membrane-based technologies [3,105,106]. Meanwhile, piezomagnetic materials remain to be developed for force sensing during needle insertion [3]. Some control algorithms such as neural networks, general bilateral control and the modified extended Kalman filter are used to reduce the measurement bias and noise [3,101,108,109,110,111,135]. 

### 3.2. Indirect Needle Insertion Force Estimation

Many authors attempted indirect methods to acquire force information during needle insertion, which include calculation methods [60,61,70,73,77], image-based methods [27,62,65,71,75,81,83,84,98,99,102,104] and actuator input methods [45,67,70]. 

In the calculation methods, scholars acquired the force and calculated the unknown force using force functions [60,61,70,73,77]. The method is available for forces that are not easy to detect. However, the instrument structure may be complex [60,61,70,73,77]. 

The image-based method is suitable for experiments equipped with imaging devices, which could reduce the cost and uncertainty of additional sensors [3,71,134,135]. They acquired the force information according to the image features such as the computed tomography (CT) value [62]. However, from the acquired image in one plane, we cannot precisely analyze the forces in the space. 

In the actuator input method, the joints of a manipulator are driven by the actuator, and the motor’s input could be directly related to the magnitude of the generated force or torque [3,108,109,110,111]. The force or torque is then acquired with the control algorithms [45,67,70,108,109,110,111]. The method is beneficial for online measurement and control. However, the error compensation mechanisms must be improved to reduce the uncertainty. 

These indirect force insertion methods have been popular for a fledging period and remain to be improved in the future. New rising technologies such as deflection and position sensors to acquire force are typical examples of indirect methods [3,71,165]. The force measurements in the future can also be combined with new imaging technologies such as single photon emission computed tomography (SPECT), positron emission computed tomography (PECT), and photoacoustic tomography (PAT). In this way, the force measurement and estimation system could achieve higher accuracy compared to the present techniques, and we can choose the available imaging technology according to the various features of the techniques and applications. 

The indirect methods can reduce the dependence of the sensor and verify the effectiveness of sensors. Therefore, the limitation of the sensors such as high cost, bandwidth limitations for feedback, instability, and HPHT sterilization could be eliminated. However, the acquired force cannot precisely describe the force details. For example, the estimation error could be obvious compared to direct force measurement methods [62]. 

## 4. Influence Factor of Needle Insertion Force

In this part, we review the parameter influence factors of the needle-tissue interaction force during the insertion process to better understand the needle-tissue interaction force model and then precisely realize force control. After a survey and analysis of research articles, we divide the factors that influence the needle-tissue interaction force into three main parts: needle properties, tissue characteristics and insertion methods. The analysis of the influence factors are summarized and discussed in Table 6.

### 4.1. Needle Properties

The properties of the needle, such as diameter, tip type, sharpness, lubrication, and cannula may affect the insertion force. 

First, we could conclude from the previous articles that a larger needle diameter could produce larger punctures as well as larger cutting and friction forces in vivo and in silicone [76,82,117,126]. As the diameter increases, the tip type effect is also amplified, and the peak axial force increases sharply [76,117,126]. 

Second, the basic tip shape could affect the insertion force in different tissues and materials. However, in various materials, the peak force was created in different tip types [76,117,126]. The reasons remain to be resolved. In addition, the inclination and normal rake angles would cause higher cutting forces in series [78,79,120]. The increased cutting edges would also lead to lower friction forces in artificial materials [68]. The bevel angle is another important parameter that affects the magnitude of the force. Lower bevel angles can cause lower axial forces and smaller deformations in almost all materials and tissues [25,75,92,122]. In addition, the total force could be influenced by the asymmetrical tip considering the insertion orientation and the fiber trend of inhomogeneous materials [12,62,83]. 

Sharpness and lubrication are other important influence factors on needle-tissue interactions. However, from the related articles [56,134], we could speculate that the effects of sharpness and lubrication may be synchronously affected by diameter, and the threshold values of diameter remain to be further researched. However, the lubrication independent of diameter, type and manufacturing affect the insertion force in polyurethane membranes [56,134]. Finally, cannula and trocar can affect the needle-tissue contact area and cutting force [66,81,92,95]. However, research specially focusing on the effects of cannula was not found in public articles.

### 4.2. Tissue Characteristics

In this part, we introduce how tissue characteristics affect the needle insertion force, such as material or tissues, experimental pretreatment and individual differences.

The most popular materials during insertion experiments can be divided into artificial materials and living tissues [25,27,28,42,44,69,74,75,76,119,122,126,127,128,129,130]. The porcine and bovine tissues or organs are most commonly used in insertion experiments [25,27,28,42,44,69,74,75,76,119,122,126,127,128,129,130]. Meanwhile, many scholars presented artificial phantoms to replace the use of animal organs considering reproducibility, visibility, ethics and morals [22,46,82,122,133,134,135,136,137]. Although manmade materials have many advantages, we must make a reasonable selection in accordance with practical considerations owing to the differences between artificial materials and practical organs. From microscopic observation, the needle-tissue insertion force between artificial and real biological tissues can vary due to the significantly different microstructures [38,72,76,122,138]. From public experimental data analysis [38,72,76,122,138], some important indexes such as the force-displacement linearity, frictional coefficient, motion formation influence, rupture toughness, elasticity modulus and crack shapes are obviously different during needle-tissue insertion. The manmade material replacements introduce considerable uncertainties for research in terms of the unknown suborgan and pathological changes in practical tissues [15,28,74,82,112]. In recent years, researchers began to focus on in vivo experiments on human or porcine organs [15,28,74,82,112]. 

The interaction forces between animal and human tissues are not definite. In insertion experiments of skin, the insertion force of porcine skin is larger than that of humans [127]. In blood vessel insertion experiments, the force–time curve is almost the same for humans and rabbits [128]. In [129], the stiffness force of the retinal vein in vivo is much higher than ex vivo. From that, we can infer that the stiffness force may be influenced by whether the tissue is alive or dead. On the other hand, the time between death and the experiment could also affect the magnitude of the axial force [130]. In [28], it was found that the presence of skin could clearly influence the peak force. 

In clinical applications, additional factors such as blood flow, tissue interlace, moisture, temperature and patient-specific criteria such as age, gender, BMI, ethnicity, prior treatment, stage of cancer, and Gleason score affect the interaction force and must be considered [27]. 

### 4.3. Robot-Assisted Insertion Method

Here, we review the needle insertion methods with varying speeds, motion modes, drive modes and locations, which will affect the insertion force during puncture. 

Between materials, the relationship between force and speed could be different. As a whole, there is a negative correlation between the insertion velocity and the axial force in certain conditions [74]. When the speed increases to a threshold value, the total force remains nearly the same [25,76]. We could conclude from previous articles [25,76] that the threshold value could be determined by multiple parameters such as the properties of the needle, the materials and the interaction mechanism between them. We ought to highlight that a larger speed could lead to a larger slope of the force–displacement curve in artificial materials, whose linearity is significant for us to establish a linear insertion control system [116,119]. 

Except for a series of analyses on the force–velocity relationship, we could pay attention to the various motion formations resulting in different insertion forces. Either the total axial force or the simple friction force could be reduced by between 10% and 150% with the help of the rotation in both animal tissues and artificial phantoms during insertion or extraction [116,118,124,131]. Meanwhile, the vibration and twisting-rotating motion could also affect the total axial force [67,90,123]. 

From the point of view of the drive mode, robot-assisted needle insertion is much more stable than manual manipulation and could maintain a constant speed, as expected. From the publicly available experimental data, the magnitudes of the forces during automatic and manual operation are different in various tissue or materials [5,25]. Interruption during insertion can lead to a larger insertion force [25]. 

As discussed in previous sections, the tissues are famous for their inhomogeneous and anisotropic properties. Thus, the insertion location can also affect the axial force [115,144]. 

## 5. Parameter Identification for Needle Insertion Force Control

Although we adequately review the influence factors, the parameters must be different in different stage for unknown soft tissues or phantoms under different conditions. Therefore, the robot manipulator must be able to handle unknown conditions. For this reason, we analyze the parameter identification methods during needle insertion to keep track of the unknown parameters in this section. 

### 5.1. Offline Parameter Identification

Some commercial software was used to construct complex models and the parameters directly. Asadian et al. [55] identified the undecided parameters to establish whole force modeling with MATLAB by attaching functions and the system identification toolbox. They regarded each function as a nonlinear state-space gray-box model and presented an iterative prediction-error minimizer and an adaptive Gauss-Newton search method to realize parameter identification. Considering a mass of data from the statistical method, Podder et al. [27] used the statistical software package “SWstat+” to formulate the models in accordance with all procedure-specific and patient-specific criteria. 

Some scholars processed experimental data and then constructed functions or models. Simone [23] fitted each experimental datum point by point to acquire unknown parameters and then found the complete insertion force modeling with mean values of the parameters. Especially in [22], the authors established whole force modeling by identifying uncertain parameters using sinusoidal signals at different frequencies and speeds. Yan et al. [46] recognized patient-specific factors in a statistical sense with a backward stepwise regression method and established a baseline model considering all of these parameters. 

### 5.2. Online Parameter Identification

The least squares method is the most common online parameter identification method in needle insertion [15,29,60,72,137]. Barbé et al. [15] presented a two-parameter model related to needle-tissue interaction forces. They used the robust recursive least squares method to estimate the models. DiMaio and Salcudean [29] provided the least squares method to identify the unknown parameters online in FEM-based modeling. However, the work shows poor real-time performance due to the excessive calculation of FEM as shown in Section 2. From the cell perspective, Xie et al. [137] identified the parameters of the supposed second-order polynomial model with the least squares method. Boroomand et al. [72] presented a LaGrange-based dynamic model of a coupled needle/tissue system with an output of the needle deflection. They realized real-time identification with a least squares parameter estimation method. 

Fukushima et al. [60] used a recursive least squares method and a disturbance observer to acquire the total force of coaxial needle and estimate the friction force. Asadian et al. [45] used the LuGre model to describe the friction forces during all phases of the needle-tissue interaction. Here, they presented sequential extended Kalman filtering (EKF) to detect the parameters of the analytical model efficiently. 

Although system identification technology is relatively successful in artificial intelligence (AI), this technology is still in its preliminary stages in the area of MIS, especially for robot-assisted needle insertion force control. The above articles are the only works in areas related to robot-assisted needle insertion force control. Therefore, we sum up some typical parameter identification methods in related areas to apply these methods to robot-assisted needle insertion in the future. We thus better realize precise and stable control according to [53,80,143,144,145,146,147,148,149,150], as shown in Table 7. Some parameter identification methods closely related to the features of the models and control schemes are not discussed here [144,145,146,147,148,149,150].

## 6. Robot-Assisted Needle Insertion Force Control System

In this section, we focus on the topic of needle insertion force control. The fundamental force control algorithms are classified in Table 8 according to the relationship between position or velocity and applied force, the application of direct force feedback, or their combinations. In the first part, we will review the most relevant articles in detail according to the classification. Considering that the force control of needle insertion is still in its initial stages, in the second part, we also include some related topics in concerned areas to enlighten more scholars to enhance the accuracy and robustness of the force feedback and control algorithms. 

### 6.1. Robot-Assisted Needle Insertion Force Control System

The proportion (P), proportion integration (PI), proportion differentiation (PD), and proportion integration differentiation (PID) algorithms are common force control algorithms [17,18,152]. Lee et al. [18] provided a repulsive force feedback control based on a dynamic model of the electrorheological (ER) haptic master to enable the surgeon to feel the force feedback during robot-assisted MIS. In this work, they provided a Kalman filter to maintain the stability of the force feedback signal. The force feedback would result in the P controller producing an available intensity of the electric field reacting in the ER fluid domain of the haptic master, and then, the surgeon would feel the stiffness of the materials and tissue. 

Graña et al. [152] provided a haptic platform based on electrorheological fluids (ERF) for surgical needle insertion to improve surgical skills and preoperative planning with a PI force controller considering stability compared to the PID controller. 

Kim et al. [17] established a haptic device to realize the sense of touch. In this work, the master robot completed the expected force according to the supposed model with the aid of a PID controller. First, the open-loop controller was designed to obtain the control voltage of the haptic master motor. The PID controller then regulated the remnant force in accordance with the control voltage determined by the difference between the desired force and the output. After that, the current driver receiving the control voltage controlled the input current for solenoid coils of the bidirectional clutch (BDC) with the purpose of finding an available magnetic field. Thus, the viscosity transformation of the magnetorheological fluid (MRF) by a magnetic field would produce a torque acquired by a torque transducer translating into a force with a conversion vector. In the practical application, an improved model reflecting energy dissipation gradually rather than instantaneously as shown in this work would result in higher error due to the magnetic properties of the MRF. 

Some authors focused on hybrid force control in a surgery robot [20,153]. Jayender et al. [153] provided a 7-DOF robot manipulator with a hybrid impedance control scheme to realize synchronous force/position control for performing catheter insertion to achieve less vessel damage and ensure surgery security during angioplasty. In this work, the authors showed two control loops—the outer loop produced the online position and force profiles tracked by the desired torques for each of the links, whereas an expected Cartesian acceleration of a joint was generated in the inner loop. 

Wells et al. [20] provided a hybrid position/force control to realize sub-tactile force range manipulation for a handheld robotic system to reduce tissue damage and enhance human tactile feedback. In this work, the authors implanted a control algorithm with the advantages of micron-fine position control and the FBG force sensor by obtaining the tip’s interaction and answering with an opposing tip movement to avoid the disadvantages of tactile feedback and depth perception. Reductions of 42% and 52% were achieved for the mean and maximum forces, respectively, for vitreoretinal microsurgery with the aid of the control algorithm. In the condition without force, the system response with position control was combined with a low pass Butterworth filter. Once any constraints exceed the threshold value, the force control loop would work, and the output position would be regulated. In addition, the anisotropic force control was also presented to distinguish the differences between soft and hard materials. 

**Figure 9:**
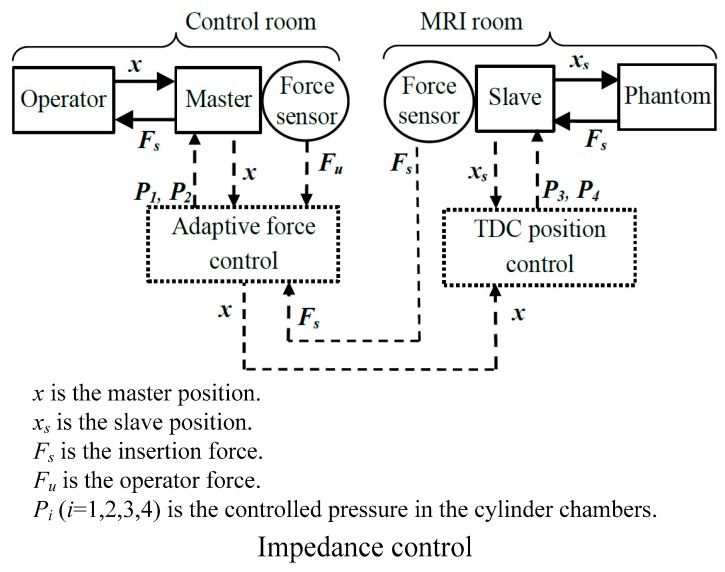
Control architecture of the master-slave system: solid lines represent physical interactions; dashed lines represent signals [102].

Some scholars focused on force control considering the safety of the operator under a radioactive environment [65,81,102,104]. Franco and Ristic [102] provided a control scheme for needle insertion under the guidance of magnetic resonance imaging (MRI) to realize laser ablation of liver tumors. They provided precise position control of the slave with a time delay control (TDC) scheme and adaptive force control of the master compensating the actuator’s friction. The control architecture is shown in Figure 9. 

Piccin et al. [65] presented a new insertion manipulator with the use of CT equipment to reduce X-ray exposure and increase the accuracy and efficiency of the surgery. The authors presented a new chuck providing an abundant space to avoid the effects of tissue fracture when the grasping needle is passively inserted into the soft tissue. In this way, the force acting on the needle could be acquired, and long-distance force feedback is established. In this work, the authors presented a three-channel bilateral controller with force environment compensation supported by related articles [104]. In this work, they modified its dynamics with the position control loop on the master side. Meanwhile, the force-feedback transparency was increased by the force control loop at the master side. 

Buzurovic et al. [81] presented a new robot-assisted brachytherapy system based on image guidance to enhance the precision of needle motion. In this work, two predictive control strategies were provided to improve the insertion efficacy and system dynamics. In the first part, they predicted insertion force with neural network predictive control (NNPC) with a theoretical linearized state-space model. With the help of the nonlinear model of the robotic system, the controller acquired the control input vector (motor voltages) to achieve optimal performance according to the previous predicted performances and the force of the future system over a finite time interval. The experimental results showed that the control system was able to maintain robustness even with a high force gradient under complex dynamic behavior. In second part, the authors utilized feed-forward model predictive control (MPC), enabling the controller to instantly compensate the effect of the acquired disturbance’s impact instead of waiting until the effect was performed, considering the feedback control for the contact force. Compared to the MPC method, NNPC did not consider the insertion force and compensate the displacement by decreasing the force gradient. In this work, the authors implemented passive insertion force control accompanied by position and velocity control and considered the measured and unmeasured disturbances. Meanwhile, procedure- and patient-specific criteria were considered in the proposed MPC method. 

### 6.2. Related Force Control Algorithm

A cell is a special soft tissue; cell injection requires more accurate needle insertion force control algorithms due to the small sizes of cells. From the soft tissue injection perspective, we will review some articles to better guide the future work on automatic needle insertion [142,151]. 

Xie et al. [142] presented a force control method to regulate the penetration force in an expected trajectory to realize automatic cell injection in a robot-assisted cell microinjection system. The proposed control scheme comprised two control loops. The outer loop was a nonlinear force-tracking controller that considered feedback linearization, whereas the inner loop was an impedance control that specified the needle-cell interaction. Huang et al. [151] presented an automatic cell injection system for batch-suspended cells to enhance the success rate and reduce expert training. In this work, the authors estimated the injection force online with a polyvinylidene fluoride microforce sensor attached to the micropipette. They then acquired an experiential relationship between the injection force and the expected injection trajectory with calibration. Later, the authors decoupled the out-of-plane cell injection into a position control in the X-Y horizontal plane and impedance control on the Z-axis. After that, they manipulated the injection pipette with a position and force control algorithm.

It is worth noting that the needle-tissue interaction via the impedance control and utilized the real-time force control to determine the needle position. It is critical for the force control system to maintain stability and accuracy during needle insertion. However, disturbances such as breathing motions would lead to some errors during surgical procedures; these have attracted increasing attention [154,155,156,157,158,159,160,161,162]. Some authors focused on this topic as follows. 

Moreira et al. [162] selected the Kelvin-Boltzmann force model considering its accuracy and feasibility compared to other candidate models and found a force control scheme with active observer (AOB). They then demonstrated the role of the viscoelastic model in the interaction force control scheme and assessed the stability and robustness of the control scheme in theory and in vitro experiments. In this work, the force control under moving conditions and physiological motion compensation were estimated. The compensation ratios for the breathing and heartbeat motions are 87% and 79%, respectively. Similarly, Zarrad [161] provided an AOB in teleoperation to provide force feedback to the surgeon. 

**Figure 10:**
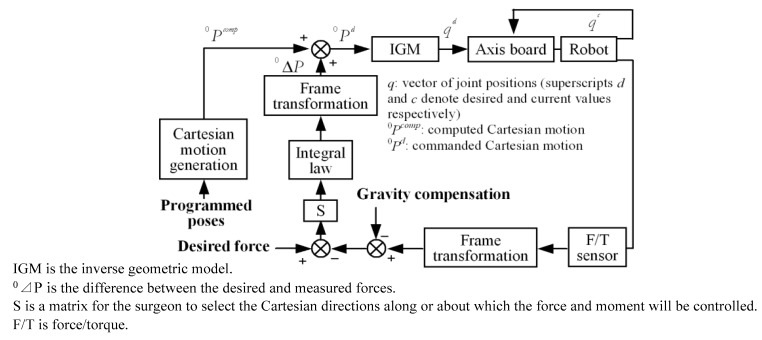
External hybrid force-position control scheme [160].

Dombre et al. [160] presented an external hybrid control combining position and force and composed of two embedded control loops to maintain constant exerted force between the dermatome and the skin in a homogeneous skin-harvesting robot. The external hybrid force-position control scheme is shown in Figure 10. 

A series of authors attempted to offset the motions and keep the robot stable [154,155,156,157,158,159]. Pierrot et al. [154] manipulated an ultrasound probe interaction with a human body by applying an external force control in the Hippocrate robot. Dominici et al. [159] provided predictive force control with the aid of a process model to speculate future behavior based on current and previous forces. Lagerburg et al. [155] developed an automatic tapping device instead of pushing, which could be useful for tissue movement due to problematic needle insertion. Zemiti et al. [156] implemented a damping force control by calculating the expected velocities according to the exerted force in a laparoscopic surgical robot. The control scheme was famous for its force sensor-less implementation. Cagneau et al. [157] presented an iterative learning control (ILC) combined with classical damping control, which has the advantage of compensating periodical breathing motions in a laparoscopic surgical robot. Zarrouk et al. [158] implemented a feedback force control based on model reference adaptive control to acquire the contact efforts on a heart surface. However, the supposed control scheme was estimated only by simulation without considering the noisy characteristics. Xue et al. [166] presented a force control system including two loops in order to match the force functions from slave robot, the inner loop of which is a current controller and the outer a force-tracking controller. In this way, both the precision and the speed could be guaranteed and the methods could be suitable to all haptic interfaces with force sensing.

## 7. Discussion

The operating environment of the surgical robot can be a biological organ or a soft tissue with completely different physical properties. Its complex operating environment such as different pressure changes, temperature changes and humidity environment results that its mechanism and control design must be targeted and adaptive design. It is of great theoretical and practical value to study the tissue mechanism and control methods of surgical robots with different motion ability to adapt to different environmental conditions.

Several typical models for describing biological tissue are linear elastic model, nonlinear elastic model, linear viscoelastic model, nonlinear viscoelastic model and biological heterogeneity and homogeneity model. In robot surgical system, to meet the requirements of real-time computing, we often make the constitutive equation for simplification. Therefore the future study would be more focusing on linear elastic model, linear viscoelastic model with the isotropic property.

Without a reliable evaluation system of accuracy, there is no way to define the precision of modeling from the quantitative point of view. There are two basic verification methods. The first one is the convergence of model simulation results. This method was used by most early researchers. The second one is compared with the actual biomechanical experiment results. This method has better reliability, but there are few studies in this area, and no quantitative evaluation system has been established. Therefore, the establishment of reliable means to verify the accuracy of the model is one of the future direction of development.

Due to the small size of the surgical robot, the micro-sensor should be based on MEMS technology while the size of sensitive element is under micrometer.

We use a variety of integrated bit sensor for real-time monitoring to achieve sensor technology. The general wired network sensor to the operation of real-time detection has brought some inconvenience since it is easy to be coiled with the other instrument. Wireless network sensor is a good solution to this problem. In a variety of complex environments, they are able to successfully collect data, which is a qualitative leap for the development of surgical instruments.

Intelligent sensor is actually refers to the soft sensor technology, which is mainly used in the sensor artificial neural network, fuzzy reasoning and expert systems and other advanced technology to make it more humane. The microprocessor system is expected to be added to the intelligent sensor. With the system collecting data processing, the surgeon can use the computer to obtain real-time acquisition results. In the future development of the sensor, the technology will become a key aspect in surgical robots.

Faced with dynamic change, unknown and complex external environment, the accurate perception of the environment is the basis for decision-making and control. Perceived information fusion, environmental modeling, environmental understanding and learning mechanism will be the important part of environmental perception and control strategy in the future.

In the face of dynamic changes in the external environment, surgical robots must be based on established operational tasks and environmental perception results with the built-in algorithms for planning, decision-making and control to achieve the ultimate goal. In the absence of human intervention or large delay in the case, autonomous control can ensure that the risk of surgery to complete the task would be avoided.

## 8. Challenges and Future Work

As shown in Table 2, from the application of needle insertion, the FEM, energy, statistical and analytical methods all could be used in needle deflection or tissue deformation, path planning or navigation and force analysis. Initially, with the help of machine learning, the FEM could also be used for online control featured with accurate representation of complex geometry and material properties. But in the future, the research need to reduce the time delay caused by excessive calculation and keep a balance between the control precision and calculation speed. Secondly, with the help of novel advanced control methods, the energy methods could online reflect energy variation and detail information. Thirdly, with the help of big data analysis, the statistical methods could acquire data distribution characters and reflect patient-specific and procedure-specific criteria in the future. The huge workload of data correlation analysis would be reduced.

As shown in Table 3, the novel analytical modeling methods should be developed in the future. From the analysis of the table, the following rules should be taken into consideration. Firstly, the new stiffness force model could better describe the nonlinear force caused by small or large deformations. The parameters could reflect the dissimilar material properties and capture the local effects or represent the total solution. In this way, the time of membrane rupture could be precisely predicted and the online force control could be better predicted. Secondly, the new friction force model should reflect both dynamic and static friction. At the same time, the model should avoid the influence caused by special needle or material properties with low error. As the needle-tissue relative velocity and presliding displacement in a dynamical condition are hard to be collected, the novel method should be without acquiring these parameters. Besides, the online force distribution of the needle during insertion could be also studied with the help of the novel electronic skin. Finally, the cutting force should be further studied in order to reflect real-time force property wholly. Meanwhile, to better study the cutting force, the further study should focus on the effects of the contact areas and resistances, reflect tip characteristics and the tearing or puncturing processing. In this way, the surgical process could be better understood and the delay time would be shortened.

As shown in Table 5, we can design a novel transducer that takes into consideration measurement ranges, resolution, sensitivity, sterilization and frequency response in limited spaces and in multiple DOFs in order to acquire online force perception and control in the future.

The future study should have a balance between the increasing DOFs and the decreasing space. On the one hand, the DOFs reflect details regarding needle insertion force in different directions, which are the key parameters for sensor design. According to our review of public articles, the DOF is only one in the early time and recently the DOFs of needle improve to 6 or 7 in different applications. However, the size of the sensor needs to be diminished further, once the DOFs improve. On the other hand, the space limitation and weight must be taken into consideration because of the narrow scope for both operation and portability. With novel materials and MEMS technology, the smaller sensors could also be developed. Furthermore, the novel electronic skin technology could also be studied with MIS technology, so the distribution of the interaction force during the insertion process could be monitored. At the same time, the sensor design of online force visualization could be a key technology to break through in the haptic study of MIS. In the end, the multi-perception fusion could be further developed with help of imaging and haptic technology.

As we all know, transducers are required to perform in wet and hot environments; they must be made up of biocompatible materials in accordance with the sensor environments during needle insertion. We must therefore design the available waterproof sensors, without any cracks disturbed by tiny bioactive substances. On the other hand, good sealed sensors can also be developed. When it comes to biocompatible materials, we can design an appropriate composition of sensors in the light of no reaction as well as safety with the body, easy acquirement and low cost. In clinical applications, sterilization is necessary during surgical operation in conjunction with physics and chemistry methods to protect patients from microbial infections. As such, these sensors must be able to work well after sterilization processes, such as chemical erosion, HPHT or EMI. Finally, the soft materials for haptic sensing could also be developed because of the flexible manipulator.

After the application period, the operational environment may have altered the transducer’s stability. If we use a sensor over a long period or under conditions that are difficult to change, we could develop convenient and precise calibration methods in order to ensure the stability, reproducibility and accuracy of force sensing.

In the analysis of the influencing factors of the mechanical model, we construct the premise of soft tissue mechanics, such as linear elasticity. However, the soft tissue tends to be viscoelastic or superelastic. For the more accurate simulation of the puncture process, the viscoelastic properties of the soft tissue should be taken into account and the nonlinearity would be studied. In future studies, we would consider the microscopic and macroscopic analysis of the factors affecting the puncture force, such as the characteristics of the puncture force from the perspective of fracture mechanics. 

As shown in Table 6, the correlations between various factors and models are not studied or inconclusive. In the future study, these correlations could be further studied in order to better establish the models. Meanwhile although many scholars have analyzed the various factors of puncture force, the influence of each factor is mutually restricted. In the future study, a number of influencing factors should be analyzed statistically and the weighting factors are put forward to obtain the optimal combination of parameters. This will lead to the establishment of a more optimized mechanical model, while optimizing the control strategy of needle. 

The existing research is mostly based on the single organ, while the actual human structure and constraints are complex and changeable. This makes the calculation and simulation accuracy limited in some degree. Therefore, in future studies, the development of anatomical multibody system will help to improve the accuracy of the analysis of factors affecting the puncture force.

The purpose of parameter identification and feedback control is to enhance the system’s ability to work under uncertain conditions. If the robustness of the feedback control is strong, the system identification accuracy is reduced with the operation time reduced and the response speed improved. Therefore, the integrated method of parameter identification and feedback control will be more advantageous than individual parameter identification. It is very promising to use the feedback control to assist in the realization of parameter identification.

Force control technology are the most important factors affecting the treatment effect. Based on the role of the doctor in the control loop, the force control of insertion can be divided into 2 directions to be developed. One is the way of teleoperation. The master and slave manipulator system are used. That is the doctor operates the master manipulator, and the needle is manipulated by the slave. The other is self-control, using image and force feedback information to complete the force control independently. Therefore, the autonomous control is the ultimate goal of robot assisted insertion.

Because needle insertion requires high accuracy, safety and stability, the traditional control theory is difficult to be directly applied to the analysis and design for surgical robot control. While the current research in this area is still in an initial stage. The problem is mainly caused by the uncertainty of needle insertion control. That is the uncertainty of soft tissue attributes, the noise interference of medical images, the accuracy and delay characteristics of force control. In the future, the force control could be updated with the improvement of the above three topics.

From the overview of the review, we hope that future research can make a breakthrough from the following aspects:

1. The establishment of virtual surgery model.
2. Delay problem caused by the ultra-long distance.
3. The enhancement of human-computer collaboration and the system transparency.
4. The abundance of information collection and feedback in the focus area.
5. The improvement of the apparatus space movement.

## 9. Conclusions

The advancement of force feedback and control will help us not only reduce tissue deformation and needle deflection but also provide the operator with a direct sense of operation and control over the surgical instruments during surgery. Furthermore, when the surgical robot interacts with its environment, the robot could autonomously identify the unforeseen circumstances and implement the appropriate algorithms. Due to robot use, high-powered needle insertion, even in an MIS surgery system, would be widely used with better reliability, higher accuracy, less pain, lower cost, faster recovery, and higher satisfaction. 

In this survey, we systematically summarize the key technologies from the entire process of robot-assisted needle insertion. First, the force modeling of needle insertion into soft tissue with analytical methods was reviewed. The insertion model reflects the robot environmental interaction characteristics. We then focused on the direct and indirect force measurement methods during needle insertion, which provide real-time force feedback information in a closed force control loop. The factors that influence the interaction force were discussed. Later, we focused on the parameter identification methodology to accurately accomplish intact force control during needle insertion. Finally, we reviewed the force control methods in related areas. All studies show that the force control during needle insertion is still at its initial stage. The non-axial force control is rare in all the studies. The influence factors of modeling are comprehensively studied. However, many questions remain open for investigation, especially the topics concerning the influence of tissue characteristics. The modeling could be acquired with the current direct or indirect sensor techniques. Besides, needle deflection or path planning are major problems for accurate needle control in the future research.

In the future, higher efficiency and precision algorithms would be developed with the sharp development of software and hardware, resulting in preferable sensors and identification methods. The new force modeling and control algorithms could utilize some other easily obtained physical parameters instead of force measurements and parameter identification. On the other hand, the advancement of force control is progressing, but it is still lagging behind the studies on surgical route planning and guidance using imaging technology. This may be due to the safety of operation. Needle insertion force control is still an emerging area with many problems to be resolved in the future, such as telesurgery requiring a sense of immediacy, the combination of touch and vision sense with progressive emergency processing technologies, the portability and miniaturization of surgical and detection instruments, the microforce control and multi-DOF manipulation in tiny anisotropy materials and tissues. 

## Figures and Tables

**Table 1:** The article group of the survey, reflecting the correlation for the related topics.

Group	Topic	References
G1	Methods of needle insertion force modeling	[4,12,14,15,17,22,23,24,25,26,27,28,29,30,31,32,33,34,35,36,37,38,39,40,41,42,43,44,45,46,47,48,49,50,51,52,53,54,55,56,57,58,59]
G2	Needle insertion force measurement	[12,15,16,27,28,29,40,41,43,45,46,50,60,61,62,63,64,65,66,67,68,69,70,71,72,73,74,75,76,77,78,79,80,81,82,83,84,85,86,87,88,89,90,91,92,93,94,95,96,97,98,99,100,101,102,103,104,105,106,107,108,109,110,111]
G3	Influence factor of needle insertion force	[5,25,27,28,40,42,43,44,49,66,67,68,69,74,75,76,78,79,90,92,112,113,114,115,116,117,118,119,120,121,122,123,124,125,126,127,128,129,130,131,132,133,134,135,136,137,138,139]
G4	Parameter identification for needle insertion force control	[15,19,22,23,27,29,46,53,55,60,72,80,101,140,141,142,143,144,145,146,147,148,149,150]
G5	Robot-assisted needle insertion force control	[13,17,18,20,21,65,81,102,104,151,152,153,154,155,156,157,158,159,160,161,162]

**Table 2:** Methods of needle insertion force modeling.

Method	Advantages and Limitations		Applications				References
			Needle Deflection & Tissue Deformation	Path Planning & Navigation	Force Analysis	Online Force Control	
Finite element method	Accurate representation of complex geometry; Inclusion of dissimilar material properties; Capture of local effects	Excessive calculation and high precision mostly rely on their inputs; In vivo and online are not available	√	√	√	×	[4,29,31,40,44,47,49]
Energy method	Calculate energy variation from deformation; Easy representation of the total solution; Available for complex motion forms	Neglect the specific process; Does not reflect online detail information	√	√	√	×	[17,38,39,59,114]
Statistical method	Acquire data distribution characters; Reflect patient-specific and procedure-specific criteria; Data correlation analysis	Require high integrity; Huge workload; Offline estimation	×	√	√	×	[27,46]
Analytical method	Reflect locally and totally; Not limited to its boundary conditions; Fast computation; Online estimation	Complex formation; Not in detail	√	√	√	√	[12,14,15,22,23,24,25,26,28,29,30,31,32,33,34,35,36,37,43,45,48,50,52,53,54,55,56,57,58]

In the column of Applications, “√” means yes, “×” means no.

**Table 3:** Analytical methods of needle insertion force modeling.

Modeling	Method	Advantages and Limitations		Applications				References
				Needle Deflection & Tissue Deformation	Path Planning & Navigation	Force Analysis	Online Force Control	
Stiffness force	Nonlinear spring model	Describe the nonlinear force caused by large deformations; Inclusion of dissimilar material properties	Higher root mean square error; not reflect online detail information	√	√	√	√	[23,24]
	Quasi-static model	Lower root mean square error; Capture of local effects; Easy representation of the total solution; Available for complex motion forms	Neglect the specific process; the specific offline parameters used only for corresponding conditions	√	√	√	√	[23]
	Hunt-Crossley model	Consider the penetration depth; match the deformation caused by needle insertion well	Neglect small motions between two objects; require high integrity; huge workload	√	√	√	√	[37]
	Exponential model	Reflect locally and totally in detail; lower root mean square error; fast computation; online estimation	The specific offline parameters used only for corresponding conditions	√	√	√	√	[28,52]
	Contact model	Consider mechanical properties and deformation; avoid the influence caused by special needle and material properties	Unavailability of the online estimation methods	√	√	√	×	[25,26]
Friction force	Modified Karnopp model	Reflect the dynamic friction and static friction; Capture the subtle effects of the Stribeck effect and Dahl model in soft tissue	Within a “dead zone” near zero velocity	√	×	√	√	[23]
	Modified Winkler based model	Affect the measurement of relative velocity; reflect force distribution	Difficult to obtain and estimate the criteria of the friction models; relative movement is invisible	√	×	√	×	[29]
	Fourier series based model	Avoid obtaining the needle-tissue relative velocity	Not reflect detail information; Huge workload	√	√	×	√	[31]
	Elasto-Plastic model	Avoid significant presliding displacement in a dynamical condition.	Relative velocity is hard to obtain	√	√	√	√	[32,33]
	Relative velocity model	Instead of the absolute velocity to focus on the relationship between the friction and the velocity; distinguish high or low relative velocities	Not reflect condition in detail under low relative velocities	×	×	√	×	[34]
	Damping based model	Calculate the cutting force from the total measured force	Neglect the specific process; Not reflect online detail information	×	×	√	×	[54]
	Thickness and elastic modulus based model	Consider both the thickness and the elastic modulus of the material	The specific offline parameters used only for corresponding conditions	×	×	√	×	[56]
	Elastic modulus and real-time friction model	Nonlinear local elastic modulus and real-time friction condition	Relative velocity is hard to obtain	×	√	√	√	[42,43,45,55]
	Dahl model	Capture presliding displacement; Describe viscous friction in low-velocity regimes; predict the friction lag	Cannot capture the Stribeck effect and reflect the static friction.	√	×	√	×	[36,163]
Cutting force modeling	Constant model	Simple; Easy to calculate	The specific offline parameters used only for corresponding conditions	×	×	√	×	[12,22,23,54]
	Deformation phase based model	Reflect real-time force property	The force is only acquired during deformation	√	√	√	√	[50]
	Maximum cutting force model	Consider the effects of the contact areas and resistances; reflect tip characteristics and the resistances; reflected the tearing or puncturing	The specific offline parameters used only for tearing or puncturing	√	√	√	×	[56]

In the column of Applications, “√” means yes, “×” means no.

**Table 4:** Spring-damper based force modeling.

Model	Legend	Formula	Parameter	References
Linear elastic model	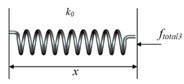	$f_{total1}(t)=k_{0}x(t)$	*t*: acting time; *x*(*t*): the deformation function; *k_i_* (*i* = 0,1,2,3,4): the spring constant; *b_i_* (*i* = 1,2,3): the damper constant; $\alpha_{1}=b_{2}/k_{2}$; $\alpha_{2}=\frac{b_{3}k_{4}}{k_{3}+k_{4}}$; $\beta =\frac{k_{3}k_{4}}{k_{3}+k_{4}}$; $\gamma =\frac{b_{3}}{k_{3}+k_{4}}$	[57]
Kelvin-Voigt model	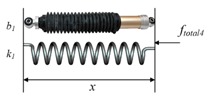	$f_{total2}(t)=k_{1}x(t)+b_{1}\dot{x}(t)$		[15]
Maxwell model	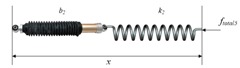	$f_{total3}(t)=k_{2}x(t)+\alpha_{1}{\dot{f}}_{total3}(t)$		[57]
Kelvin-Boltzmann model	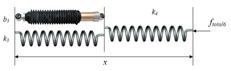	$f_{total4}(t)=\beta x(t)+\alpha_{2}\dot{x}(t)-\gamma {\dot{f}}_{total\;4}(t)$		[58]

**Table 5:** Force measurement and estimation methods for needle insertion.

Method	Technique	Degree of Freedom (DOF)		Sensitivity	Size	Cost	Advantages and Limitations		Online	References
Direct measurement method	Strain gauge	1–6	Force; Torque	Fine	Small	Low	Limited to temperature changes and electromagnetic noise; Drift and hysteresis	High strength; (Ethylene oxide or formaldehyde sterilization; Stainless protected)	Yes	[12,15,16,27,28,29,40,41,43,46,50,60,62,69,72,74,76,79,80,81,82,83,84,86,87,88,89,90,91,92,95,103]
	Piezoelectric sensor	1–3	Force	Fine	Small	High	Limited to temperature changes and charge leakages; Not for static conditions because of drifting signal	High bandwidth; High power density; Great measurement range (Stainless protected)	Yes	[68,78,85,92,100,101,107]
	Optical sensor	1–3	Force; Torque	High	Small	High	Limited to cable deformation	Works in electromagnetic interference (EMI); Reproducibility; No hysteresis	Yes	[63,64,93,94,96,97]
Indirect estimation method	Calculation method	1–7	Force; Torque	Multiple	Multiple	Multiple	Limited to a series of sensors; Complex structure	Acquires hard to detect forces	undetermined	[60,61,70,73,77]
	Image-based method	1	Force	Low	No additional space	No additional cost	Limited to experiments equipped with imaging devices; No detailed analysis of force	Works in a high-temperature and high-pressure (HPHT) or corrosive environments; Easily acquires the total force	undetermined	[27,62,65,71,75,81,83,84,98,99,102,104]
	Actuator input method	Multiple	Force; Torque	Multiple	Multiple	Multiple	Limited to uncertainties; Requires compensation mechanisms; No detailed analysis of force	Easily acquires the total force	Yes	[45,67,70,108,109,110,111]

In the column of Advantages and limitations, “( )” means the properties in part products.

**Table 6:** Influence factors of needle insertion force.

Item	Effect Factor	Empirical Value or Detail	Correlations between the Influence Factors and Insertion Force					Reference
			*F*_*stiffness*_	*F*_*friction*_	*F*_*cutting*_	*F*_*c+f*_	*F*_*total*_	
Needle property	Diameter	0.31–3.4 mm	√	√	√		√	[5,12,25,62,66,68,75,76,78,79,81,82,83,92,95,117,120,121,122,125,126,127,128]
	Bevel angle	8–85°	√			√	√	
	Inclination angle *λ*	<30°			√			
	Normal rake angle *α*	when *λ* > 70°, *α* < 10°			√			
	Multi-bevel pen needle tip	3,5	√					
	Tip type and edge	Diamond (Franseen); Beveled; Blunt; Conical; Sprotte; Tuohy					√	
		Sharpness; Lubrication; Cannula; Asymmetrical type; Manufactory	√	√	√			
Tissue characteristic	Living tissue	Human	√			√	√	[5,12,15,25,27,28,42,43,44,56,62,69,74,75,76,82,83,112,113,119,122,126,127,128,129,130,133,134,135,136,137]
		Animals: porcine, bovine, chicken, rabbit, turkey, sheep, canine	√			√	√	
		Organs: kidney, liver, heart, prostate, perineum, skin, muscle, fat, tendon, retina, vein, dura, vertebra, bone, brain, ligament, meninges	√			√	√	
	Artificial material	Polyvinyl alcohol (PVA), polyvinyl chloride (PVC), rubber, silicone gelatin, plastisol						
	Experimental pretreatment	In vivo or ex vivo; Live or dead; With or without skin Anesthetization; Moisture; Temperature; Gel elasticity; Multilayer						
	Individual difference	Suborgan or tissue interlace; Blood flow; Age; Gender; Body mass index (BMI); Ethnicity; Prior treatment; Stage of cancer; Gleason score; Pathological changes						
Insertion method	Velocity	0.0008–1000 mm/s	√	√		√	√	[5,25,28,40,42,49,67,69,74,75,76,79,90,92,115,116,118,123,124,125,131,132,139]
	Motion mode	Translational or rotational motion; Sinusoidal motion Oscillatory motion; Twisting-rotating motion			√	√	√	
	Drive mode	Interrupt or continuous; Manual or robotic				√	√	
	Direction	30°	√				√	
	Location	Suborgan or tissue interlace; Blood flow	√				√	

The blank means there is no related research or the correlation is inconclusive, “√” means yes.

**Table 7:** Typical parameter identification methods.

Item	Typical Method		Advantages and Limitations		Applications			
					Needle Deflection & Tissue Deformation	Path Planning & Navigation	Force Analysis	Online Force Control
Data-based parameter identification	System response method; Frequency response method; Correlation method; Maximum likelihood method		Acquire data distribution characters; Reflect specific criteria; Data correlation analysis	Require high integrity; Huge workload; Offline estimation	√	√	√	×
	Static system	Dynamic system						
Time-invariant parameter identification	Weighted least-squares estimation; Constrained least-squares estimation; Truncated least-squares estimation; Total least-squares estimation; Nonlinear least-squares estimation	Least-squares estimation; Ordinary least-squares estimation; Biased least-squares estimation; Generalized least-squares method; Pre-filtering method; Neural network; Wavelet network	Characterize the entire system simply	Not well reflect the real situation of the whole system	√	×	√	×
Time-varying parameter identification	Recursive least-squares estimation; Square root filtering; Reduced-rank square root (RRSQRT) filtering; Extended Kalman filtering for the estimation	Recursive prediction-error estimation; Fixed-interval optimal smoothing; Extended Kalman filtering; Neural network; Wavelet network; Radial basis function neural network; Genetic algorithm; Evolutionary algorithm; Fuzzy logic algorithm; Times series analysis method	Online control; reflect the dynamic characteristics	Improve the complexity of analysis and research	√	√	√	√

In the column of Applications, “√” means yes, “×” means no.

**Table 8:** Comparison of fundamental force control algorithms.

Algorithm Classification		Workspace	Measured Variables	Modified Variables	Modulated Objectives	Advantages and Limitations		Applications			
								Needle Deflection & Tissue Deformation	Path Planning & Navigation	Force Analysis	Online Force Control
Active stiffness control	1. Version one	Joint space	Position, force	Joint displacement, contact force	Joint stiffness matrix	See as a programmable spring; simple structure; good robustnes	Maximum controlled stiffness is influenced by the stability; require force sensor; successful in very specific tasks	√	√	√	√
	2. Version two	Task space ^a^		Position error, contact force	Stiffness matrix						
Impedance control	1. Basic impedance control	Task space	Position, velocity, force	Position and velocity error, contact force	Impedance	Direct control of the force between the end actuators and the environment; realize compliance control	Requires a lot of task planning; Need to switch between force control and position control;	√	√	√	√
	2. Position-based impedance control			Modified desired trajectory, contact force							
Admittance control			Force	Force error	Admittance	Direct control of the force between the end actuators and the environment; realize compliance control	Select appropriate parameters to ensure the stability	√	√	√	√
Hybrid control	1. Hybrid position/force	{P} ^b^	Position	Position error	Position	The position control and force control can be separately considered; Flexible to choose the strategy	Computational complexity; Location coordinates need to be determined by the environmental constraint equation	√	√	√	√
		{F} ^c^	Force	Force error	Force						
	2. Hybrid impedance	{P}	Force	Velocity error	Z_mp_^d^						
		{F}		Force error	Z_mf_^e^						
Explicit force control	PI, PD, PID, etc.	Task space	Force	Force error	Desired force F_D_	Direct force feedback	No postion feedback	√	√	√	√
Implicit force control		Task space	Position	Position error	Predefined stiffness	The position is controlled by the position for a desired force	No force feedback	√	√	√	√

^a^ Task space = {P}⊕{F}. ^b^ {P} is the position subspace. ^c^ {F} is the force subspace. ^d^ Z_mp_ is the impedance expressed in the position subspace. ^e^ Z_mf_ is the impedance expressed in the force subspace.
